# Multiple psychological senses of community and community influences on personal recovery processes from substance use problems in later life: a collaborative and deductive reflexive thematic analysis

**DOI:** 10.1080/17482631.2023.2190200

**Published:** 2023-03-16

**Authors:** Nina Kavita Heggen Bahl, Emil Øversveen, Morten Brodahl, Hilde Eileen Nafstad, Rolv Mikkel Blakar, Anne Signe Landheim, Kristin Tømmervik

**Affiliations:** a Department of Research and Development, Clinic of Substance Use and Addiction Medicine, St. Olavs University Hospital, Trondheim, Norway; b Department of Sociology and Political Science, Norwegian University of Science and Technology, Trondheim, Norway; c Norwegian National Advisory Unit on Concurrent Substance Abuse and Mental Health Disorders, Mental Health Division, Innlandet Hospital Trust, Brumunddal, Norway; d Department of Psychology, University of Oslo, Oslo, Norway; e Norwegian National Advisory Unit on Concurrent Substance Abuse and Mental Health Disorders, Innlandet Hospital Trust, Brumunddal, Norway; f Norwegian Centre for Addiction Research, University of Oslo, Oslo, Norway; g Innlandet University of Applied Sciences, Faculty of Social and Health Sciences, Section for Mental Health and Rehabilitation, Campus Elverum, Norway

**Keywords:** Community, later life, psychological sense of community, recovery, substance use problems, qualitative methodology

## Abstract

**Purpose:**

There is a pressing need for substance use services to know more about how to promote recovery from substance use problems, particularly in later life. Psychological sense of community (PSOC) is an important recovery dimension. This study aims to clarify in what ways PSOC and communities influence later life recovery processes.

**Method:**

A collaborative and deductive reflexive thematic approach was used to analyse 23 interviews with older adults in recovery from different substance use problems.

**Results:**

The findings suggest that PSOC and recovery in later life include multiple communities (relational, geographical, substance use-related, ideal and service-related) and affective states (PSOC and NPSOC). Older adults’ recovery, moreover, can be described as personal and heterogenic (with respect to community relationships, individual needs, type of substance use problem, age of onset and meaningful activities).

**Conclusions:**

The findings confirm age of onset, type of substance use problem and community memberships as essential to later life recovery. They also supplement prior evidence on community resources and challenges to later life recovery. Importantly, the new findings extend and nuance current understandings of later life recovery. Taken together, the article illustrates MPSOC as a useful concept, with central practical and theoretical implications for later life recovery.

## Introduction

For two decades now a silent epidemic of later life substance use problems has been going on in Western countries. “The baby boomers” - the largest group of older adult persons so far in many Western countries—are on the rise, also with respect to those having substance use problems (Chhatre et al., 2017; Foster et al., 2021; Gfroerer et al., 2003). This cohort lives longer compared to earlier cohorts of older adults and is likely to bring with them their substance use problems into old age (Yarnell et al., 2020). As a consequence, there is a pressing need not only for substance use services tailored for older adults but also for more knowledge about how to promote recovery in later life (Gfroerer et al., 2003; Johannessen et al., 2016; Morgan et al., 2011). Particularly, health professionals need knowledge about how to provide broader care of older adults by inclusion of significant others (e.g., family careers) in recovery (Johannessen et al., 2015; Morgan et al., 2011).

Being involved, to feel sense of belonging and have a meaningful life without substance use are key aspects of recovery from substance use problems in emerging adult and adult years (Bahl et al., 2019, 2022; Granfield & Cloud, 2001; Groh et al., 2007; Laudet, 2007; Mayberry et al., 2009; Moore et al., 2018; Mudry et al., 2019; Panel, 2007; Wenaas et al., 2021). However, we know little about these aspects of life when it comes to older peoples’ recovery processes. Psychological sense of community (PSOC) and recovery processes are age-specific phenomena (Bahl et al., 2022; Blow et al., 2000; LaBarre et al., 2021) and there are several factors of an old age complicated by substance use to consider. First, community participation—a necessity for PSOC and recovery—is often challenged with deteriorating health, comorbidity, high likelihood of depression, shame, loneliness and isolation that older adult with substance use problems often experience (Emiliussen et al., 2017; LaBarre et al., 2021; Morgan et al., 2011; Satre et al., 2004; Yarnell et al., 2020). Second, older adults in recovery processes are a highly heterogenic group with respect to age of onset and substance use problems, with consequences for which social resources and community memberships that are available.

In order to promote recovery from substance use in old age, we need to understand the promoting and challenging elements that different communities may propose in later life recovery. We need more broad and in-depth investigations of PSOC and community influences on later life recovery including a multivocality of different sub-groups of older adults having substance use problems. This study is a collaborative, deductive, and reflexive thematic investigation asking: *In what ways do older adults with substance use problems experience their communities as influencing their personal recovery processes?*

## Psychological sense of community (PSOC) among older adults with substance use problems

The concept psychological sense of community (PSOC) refers to meaning systems of care, support, trust, responsibility, social relationships, identity, and meaning (Bahl, Nafstad, et al., 2021; Brodsky, 2009; Kloos et al., 2011; McMillan, 1996; Nowell & Boyd, 2010; Sarason, 1974). Conceptually this phenomenon is predominantly captured by four dimensions, confirmed also of being part of older adults’ ordinary concepts (Bahl, 2018; Zaff & Devlin, 1998); (a) feeling of belonging and identification with the community (membership); (b) sense of having an impact on their community and experiencing an acceptable influence from the community (influence); (c) effort to contribute to the community needs while simultaneously experiencing that the community integrates and fulfils your individual needs (integration and fulfilment of needs); and (d) a feeling that the members of the community have a shared experience and a common history that the members of the community will continue to share (shared emotional connection) (McMillan & Chavis, 1986; Peterson et al., 2008).

In addition to the above four core dimensions, the concept has more recently been extended by the more recent MPSOC concept which includes multiple community references (geographical, relational and ideal) and affective states (positive and negative PSOC) (Bahl et al., 2019, 2022; Brodsky et al., 2002; Mannarini et al., 2014). Negative PSOC has been defined as a centrifugal force that symbolically moves individuals away from their community (Brodsky, 1996) and operationalized by four dimensions: (a) a need to distinguish oneself from the community and its members, an experience of being unlike other community members (distinctiveness), (b) a passive uncaring attitude towards the community and its shared events, and a trend to abstain from any activities with other members (abstention), (c) an experience that the community and its members are a source of frustration (frustration), and (d) a feeling of being extraneous, unfamiliar and unconnected to the community, its members, and its shared traditions or history (alienage) (Mannarini et al., 2014).

Findings about recovery from substance use suggest that multiple communities (e.g., family, acquaintances, local and national communities) simultaneously influence recovery in emerging adult and adult years (Bahl et al., 2019, 2022; Mayberry et al., 2009; Moore et al., 2018). Furthermore, positive PSOC can play an important role in emerging adults and adults substance use recovery (Barbieri et al., 2016; Drake et al., 2005; Ferrari et al., 2002; Jason et al., 2001; Kollath‐cattano et al., 2018; Laudet, 2008; Peterson & Reid, 2003; Stevens et al., 2010, 2012). There are, moreover, findings suggesting that several NPSOC dimensions can be central in social transitions (from recovery destructive communities) as well as in the recovery identity development that the recovery processes often entail (Bahl et al., 2022; Bathish et al., 2017; Groh et al., 2007; Mawson et al., 2015). As far as we know, there is no research on PSOC or community influence on recovery among older adults with substance use problems. In fact, there is little evidence on the role of social bonds and recovery processes in this age group. Thus, MPSOC research with older adults with substance use problems is central to gain more knowledge to promote recovery among older adults, as pointed out today a growing population around the world.

## Later life recovery from substance use problems

Within substance use research recovery has been understood as personal processes where the aim across life-span is to “recover” oneself as a person finding meaning and mattering without problematic substance use (Bahl et al., 2023; Brekke et al., 2017; Kaskutas et al., 2014; Laudet, 2007). These processes necessarily take place in contexts outside of the professional health care system (Bahl et al., 2019; Brekke et al., 2017; Davidson & White, 2007; Landheim et al., 2016;). They entail everyday social participation and meaningful activities in the person’s relational and geographical communities (Bahl et al., 2019, 2022). However, recent conceptualizations suggest that in later life several social and somatic factors may deter recovery processes: For example, decline in social networks and community participation due to death of spouse or partner, loss of friends, retirement from the work community, and deterioration in physical mobility (LaBarre et al., 2021; Morgan et al., 2011). Furthermore, evidence also suggests that older adults with substance use problems are subjected to additional factors complicating recovery: comorbidity, high likelihood of depression, feeling shame, loneliness and isolation (Emiliussen et al., 2017; LaBarre et al., 2021; Morgan et al., 2011; Satre et al., 2004; Yarnell et al., 2020).

Besides these factors, one has to consider that older adults with substance use problems are a highly heterogenic group with respect to age of onset and substance use problems. Furthermore, both age of onset and type of substance use problem have consequences for social resources and community memberships available in their recovery. For instance, older adults with alcohol use disorders (AUD) and very late-onset (VLO) (after the age of 60), tend to have higher level of education, income, life satisfaction and stability of residence compared to early-onset individuals (Schonfeld & Dupree, 1991; Wetterling et al., 2003). VLO individuals also tend to have less social support than early-onset individuals, and more likelihood of late-life social stress (Liberto & Oslin, 1995). Such a pattern is on the other hand contrary to heroin users with early onset as they tend to have more social resources compared to late onset users (Boeri et al., 2008). There is also little knowledge about the social resources of older adults in their recovery processes from having problematic medicine use (Maree et al., 2016). However, there are some findings suggesting that being female, aged 75–84 years old, living alone, having lower socioeconomic status, polypharmacy, higher pain intensity and depression scores are characteristics clearly related to medication misuse and dependence (Cheng, Siddiqui, Gossop, Kristoffersen & Lundqvist, 2019).

Today then, little evidence exists about community experiences of older adults who are in recovery processes and the variety of supportive and destructive community relationships they may have in initiating personal recovery processes, in their laps and relapses and their efforts trying to maintain health and meaningful community memberships in later life. Thus, we have adopted the broad MPSOC concept in the current investigation of multiple community references (relational and geographical and ideal) as well as affective states (positive and negative PSOC) among an older adult sample with early, late and very late onset of substance use problems with alcohol, medicine or illegal drugs.

## Material and method

Thus, this study is a qualitative collaborative and deductive reflexive thematic investigation of what ways older adults with substance use problems experience their communities as influencing their personal recovery processes. We have used a collaborative research design and reflexive thematic deductive analyses with MPSOC as our integrative theoretical framework. The analysis included three relevant perspectives: community psychological (first author), sociological (second author) and peer research perspective (third author). These perspectives were chosen to triangulate in the analysis to possibly achieve more insight into the different ways the participants experienced their communities and their senses of communities as influencing their processes of recovery from problematic substance use.

### A collaborative research design

User involvement is a central requirement in Norwegian health research (Natland et al., 2017). Although rarely undertaken, user involvement has also to be embedded in the analysis of data. This study included the perspective of individuals who were experiencing, or had experienced, recovery from substance use problems through collaboration in different phases of the research process. First, in the planning of the data collection and development of the initial interview guide, a peer support worker from the Drug and Alcohol Competence Centre in Central Norway participated as member of the planning board. This guide was later adapted to older adults by the first author and collaborators at the hospital’s Clinic of Substance Use and Addiction Medicine. Second, our sample of 23 older adult participants all had experiences of personal recovery processes from substance use problems. Third, a peer researcher (third author) collaborated with the first and second authors in the analysis. This peer researcher also had recovery experiences; having personal experience of recovering from substance use problems and working with people in recovery from substance use, as well as having education in and experience with qualitative analysis methods within this field of substance use and addiction.

### Approach to enquiry

Appropriate measures were taken to meet the American Psychological Associations standards for qualitative research (Levitt et al., 2018) and quality practice for reporting reflexive thematic analyses (Braun & Clarke, 2020, 2022). With respect to dimensions for reflexive thematic analyses, this study is deductive in its theoretical approach, epistemologically experiential as well as constructivist in its perspective. To elaborate; the study’s conceptual framework was applied deductively in the coding of the material by all authors involved in the analysis. However, the framework was not used strictly to force data into PSOC or community reference categories. It was used as a guide to code by, allowing new community types and PSOC-related aspects to be included in the construction of codes. Furthermore, the study’s orientation is experiential in its aim to give voice to older adults’ life experiences. Finally, consistent with its collaborative design and focus on how multiple senses of community and communities construct later life recovery processes from substance use, the study is constructivist in its orientation.

### Recruitment and sample

A purposeful sampling strategy was used to recruit 23 older adult participants with substance use problems in three different contexts: two urban municipalities (>20 000 citizens) and one municipality (<20 000 citizens). The samples age ranged from 65 to 80 years, with about equal numbers for those aged 60–69 (12 participants) and 70–80 (11 participants). With respect to gender, 7 participants were women and 16 men.

Different groups of staff (e.g., geriatric psychologists, staff at user organizations, and substance use treatment clinics) working with older adults having substance use problems, were included and assisted in the planning of the participant recruitment in all three contexts in 2019 (pre-Pandemic times). These staffs contacted some potential participants directly by phone and physical meetings, as well as other municipal services relevant for recruitment of additional participants (e.g., general practitioners, home nursing, low threshold offers and geriatric clinics in specialized health care). These services were contacted by physical meetings, email, phone, and newsletters inviting them in the recruitment of potential participants.

All of the participants had to meet the inclusion criteria; aged 65 years or older (consistent with definitions of old age in populations with substance use problems (Choi et al., 2014; LaBarre et al., 2021); having a substance use problem with alcohol, medicine or illegal drugs; and receiving one or several services from the municipality which they resided in. All participants received a gift card with 300 NOK (approximately 28 USD) for their participation, which could be used in a range of stores. To sum up, the sample represented a variety of community and recovery experiences (see Table I).

**Table I.** Participants’ background and community belonging.

Participant	Interviewer	Region	Health care services for persons with substance use problems	Current use of substances?	Onset of substance use problems	Way of contact	Problematic substances	Relational communities	Geographical communities	Ideal communities	Substance use related communities	Service-related communities
M66 (E1)	5	East	Housing, housing allowance, NAV, organized physical exercise, general practitioner, low threshold offer (meals)	No	Early	Employer made contact	Amphetamine/Alcohol (Polysubstance use)	Family + Friends ±	Housing from the municipality +		Work related-	Group arranged by Norwegian Labour and WelfareAdministration +
M67 (E2)	4	East	Housing, contractual early retirement pension scheme (AFP) in the public sector, earlier: three different institutions, home nursing,	No	Early	Injury/hospitalization	Opioids/Alcohol (Polysubstance use)	Family + Friends +	Housing from the municipality			Interdisciplinary specialized treatment of substance use problems (ISTS) +
M77 (E3)	5	East	NAV, disability pension,	Yes	Early	Lack of income	Alcohol	Family - Friends -	Neighbourhood -		Alcohol related -	
M71 (W4)	2	West	Contractual early retirement pension scheme (AFP) in the public sector, general practitioner, earlier: institution, NAV Assistive Technology Centre, institution	No	Early	Self-initiated contact	Alcohol/sleeping pills (Polysubstance use)	Family + Friends +	Housing (private) +	Meaningful activities	Alcohol related +	ISTS + Volunteer community: The soup car +
F68 (E5)	5	East	Housing, Specialized health care (somatic) after injury, AV, Work assistance allowance (AAP), transport service card, general practitioner, physiotherapy, Earlier: six institutions	No	Early	Suicide attempt/hospitalization	Alcohol/Morphine (Polysubstance use)	Family + Friends +				Institution/ISTS ±
M68 (W6)	2	West	NAV, Crisis centre for victims of violence and abuse from partner or family, contractual early retirement pension scheme (AFP) in the public sector, organized physical exercise, earlier: institution	No	Early	Violence in the home (partner)	Alcohol	Family + Partner - Friend +	Neighbours +	Health adapted service (housing)		ISTS +
M80 (E7)	4	East	Housing, pension (not specified), outreach service, home nursing, earlier: institution	No	Early	Recommended by family to make contact	Alcohol	Family +	Neighbours +	Work		ISTS +
M77 (C8)	1	Central	Pension (not specified), home nursing, housing, NAV Assistive Technology Centre, general practitioner, geriatric psychologist, Earlier: recovery centre, physiotherapy,	No	Late	Recommended by home nurses to make contact	Alcohol	Family ± Friends +	Neighbours +	Meaningful activity		Elder care institution +
M76 (C9)	1	Central	Pension (not specified), home nursing, NAV Assistive Technology Centre, earlier: institution, general practitioner	Yes	Very late (after 60 years)	Recommended by home nurses to make contact	Alcohol	Family ±		Meaningful activity Health and age adapted service		
F65 (C10)	1	Central	NAV, contractual early retirement pension scheme (AFP) in the public sector, earlier: organized physical exercise for chronical illness, follow-up service, centre for mapping and follow‐up	No	Late	Chronic muscular pain lead to hospitalization	Alcohol	Family ± Friends +				ISTS + Service group from the municipality +
M68 (C11)	1	Central	Pension (not specified), physiotherapy, home nursing, earlier: institution (specialized health care),	No	Late	Friend assisted in making contact	Alcohol	Friends ±		Health and age adapted service		
F68 (C12)	1	Central	Housing, contractual early retirement pension scheme (AFP) in the public sector, earlier: institution,	No	Early	General practitioner made contact	Alcohol	Family ± Friends -		Health and age adapted service		Elder care ±
F73 (E13)	4	East	Pension (not specified), organized physical exercise, general practitioner, short-term specialized treatment of alcohol addiction, earlier: institution (twice), home nursing	Yes	Late (20 years)	Injury (fall)/hospitalization	Alcohol	Family + Friends	Neighbours +			ISTS ±
M76 (E14)	4	East	Pension (not specified), non-governmental organization for persons with alcohol dependence (Norske lenker), nursing home, physiotherapy, social worker, earlier: institution	No	Early	Injury (fall)/hospitalization	Alcohol	Family + Friends (new in Norwegian)+ Former friends -				Elder care + Volunteer community: The Norwegian Chains +
F66 (E15)	4	East	Housing (municipality), psychologist, home nursing, disability benefits, drug-assisted treatment, interdisciplinary team meetings Flexible Assertive Community Treatment, NAV (economic manager), general practitioner, transport service card, earlier: institution psychiatric/substance use	No	Early: Medicine, Late: Heroin (40 years old)	Death of husband who was co-addict	Medicine, heroin, amphetamine (Polysubstance use)	Family – Friends +	Housing from themunicipality +	A place to be/available communities (open at all hours)		ISTS (poly) ±
M66 (E16)	5	East	Housing, disability benefits, drug-assisted treatment, NAV, general practitioner, earlier: institutions	No (methadone)	Early	Personal contact at NAV made contact	Heroin		Housing from the municipality + Neighbourhood +			ISTS (poly) +
M69 (E17)	5	East	Pension (not specified), NAV (management of economy), department of mental health, general practitioner, interdisciplinary team meetings home nursing, earlier: institution, cancer nurse,	No	Early (not specified, but had problems in working age)	Self-initiated contact with hospital due to suicide ideation	Alcohol		Neighbour +			ISTS + Volunteer community: Salvation Army +
M69 (E18)	5	East	Housing, drug-assisted treatment, general practitioner, old-age pension, polyclinic treatment for persons with substance use problems where alcohol is the only or dominant problematic substance, low-threshold health and care offer for those with substance use problems, social worker, interdisciplinary team, earlier: psychologist, institution	No	Early	Quit work to become clean (self-sufficient), made contact due to starvation	Heroin, alcohol, amphetamine (Polysubstance use)	Family ±	Housing from the municipality +		Former member of the substance use related communities +	
M69 (C19)	1	Central	Housing, home nursing, social worker, centre for mapping and follow‐up, district psychiatric centre, earlier: institutions	No	Early	Made contact due to suicide ideation	Alcohol, medicine (Polysubstance use)	Family -	Housing from the municipality -	Meaningful activity		
M70 (C20)	1	Central	Nursing home, pension (not specified), NAV (economic management), general practitioner, social worker, earlier: institutions (detoxification/substance use treatment)	No	Early	General practitioner referred to hospital (acute)	Alcohol	Family ±Friends +		Health and age adapted service Meaningful activity		Elder care ± ISTS +
F70 (C21)	1	Central	Home nursing, pension (not specified), psychiatric nurse, general practitioner, physiotherapy, occupational therapy, primary contact (at department of health and welfare), earlier: follow-up service	No	Late (40 years old)	Hospital made contact after operation	Medicine, alcohol (Polysubstance use)	Family + Friends				
F73 (C22)	3	Central	Old age pension, organized physical exercise, physiotherapist, psychologist, psychiatrist, general practitioner, home nursing, earlier: institution	No	Very late	After hospitalization (acute)	Alcohol	Family ±	Neighbours +			ISTS +
M72 (C23)	1	Central	Old age pension, daily social offer including physical exercise (9–14), geriatric care, general practitioner, home nursing, earlier: institution (detox)	No	Early		Alcohol	Family + Friends +		Health and age adapted community		Service group from the municipality +

+ is used when the participant speaks about the community in any way related to the four dimensions of PSOC (membership, influence, integration and fulfilment of needs or emotional connection). – is used for NPSOC related descriptions (distinctiveness, abstention, frustration or alienage). Both signs are used for communities addressed in both ways. If no sign is added, the participant did only report belonging to the community and nothing further. All communities besides from ISTS are current communities which the participant where part of when the interview was undertaken.

## Material

The study material was a verbatim transcribed interview material, collected as part of a larger national project evaluating service users’ experiences with their substance use treatment services from the Norwegian municipalities. This larger project was conducted by The Drug and Alcohol Competence Centre in Central Norway on assignment by the Norwegian Directorate of Health. The present study utilized data from the national projects second wave, aimed to generate qualitative knowledge about how older adults with substance use problems experience services from the Norwegian municipalities. The material was collected by five interviewers across three Norwegian municipalities. For transparency, Table I and all excerpts used from the material include an interviewer code (1–5). Interviewer 1 is the second author and an academic researcher; Interviewers 2 and 3 worked at The Drug and Alcohol Competence Centre in Central Norway, interviewers 4 and 5 worked at The Drug and Alcohol Competence Centre in Oslo. All interviewers had academic training in conducting interviews (5 of 5) and 3 of 5 had clinical competence in communication with individuals with substance use problems.

As the data was collected by several interviewers, a semi-structured interview guide was chosen to ensure a consistent overall structure to the interviews. However, despite the semi-structure of the interviews, they were conducted in an in-depth manner so that the participants could freely describe their experiences with their current life situation, their community relationships (e.g., with family, friends, and neighbours), and the municipal services they received. Participants were specifically asked about their background, current life situation, experiences with municipal services, relationships with family members and significant others, and how others were involved in the services they received.

### Ethical considerations

The larger national study which the study material was from, was approved by the Data Protection Officer at St.Olavs hospital in Trondheim, Norway (Reference ID: ESA 17/4211). Consistent with this approval, all participants were informed about what their participation would involve, who would conduct the interview and that the interview would be anonymized and transcribed verbatim. The informants were also asked if the interview could be digitally audio-recorded, of which 2 participants declined. These interviews were conducted by written notes. Finally, before conducting the interviews, each participant was informed that they could withdraw their consent and end the interview at any point of time. All participants signed a written informed consent before the interviews took place.

### Sociocultural context: Services and family support

The social and cultural context of this study was Norway, a Scandinavian welfare state. In the Norwegian public health care system, specialist health care services are offered at the regional level and primary health care services are organized and delivered by municipalities. Several services which many older adults depend on in their everyday life are offered by the municipality, such as general practitioner, home nursing and physiotherapy. As part of Norwegian clinical substance use treatment, individuals are first offered services by the municipality. Then, if needed, they are referred to hospital—based specialized health care services. Most of the specialized services are offered to a general grown-up population, having a wide age-range. Completing specialized treatment, the patient then returns to municipal services where recovery processes can continue. The exception is early onset heroin users. They usually receive medically assisted specialized polyclinic treatment solely. However, as some of the participants in this study, there are many older adults with substance use problems who are not in touch with substance use services until they are injured and have to go into specialized health care, which then refer them to substance use treatment. Aside from an initial excess charge of 2460 NOK per year (approximately 285 USD), all services are offered free of charge.

Generally, individualistic Northern counties have had less of a family orientation than Southern and Eastern European countries (Hansen & Slagsvold, 2015). Thus, urban older adults in Norway may hold meaning systems of PSOC and well-being which emphasize own responsibility and effort (Bahl et al., 2017). As consequence, the participants interviewed may have different experiences with their communities and social relationships compared with other Western settings as well as more market-driven health care systems.

## Analysis

Reflexive thematic analyses are reflective processes of knowledge generation (Braun & Clarke, 2019). In this study, we generated knowledge about older adult’s experiences of community influences on recovery from substance use problems from the use of collaborative deductive coding guided by the MPSOC framework. Three authors (first, second and third authors) collaborated in the analysis and used their perspectives (community psychological, peer and sociological perspective) to deductively construct codes and themes. Our approach to the collaborative reflexive thematic analyses has been, an organic, creative process consisting of seven stages of analysis (see Figure 1).

**Figure 1a.**
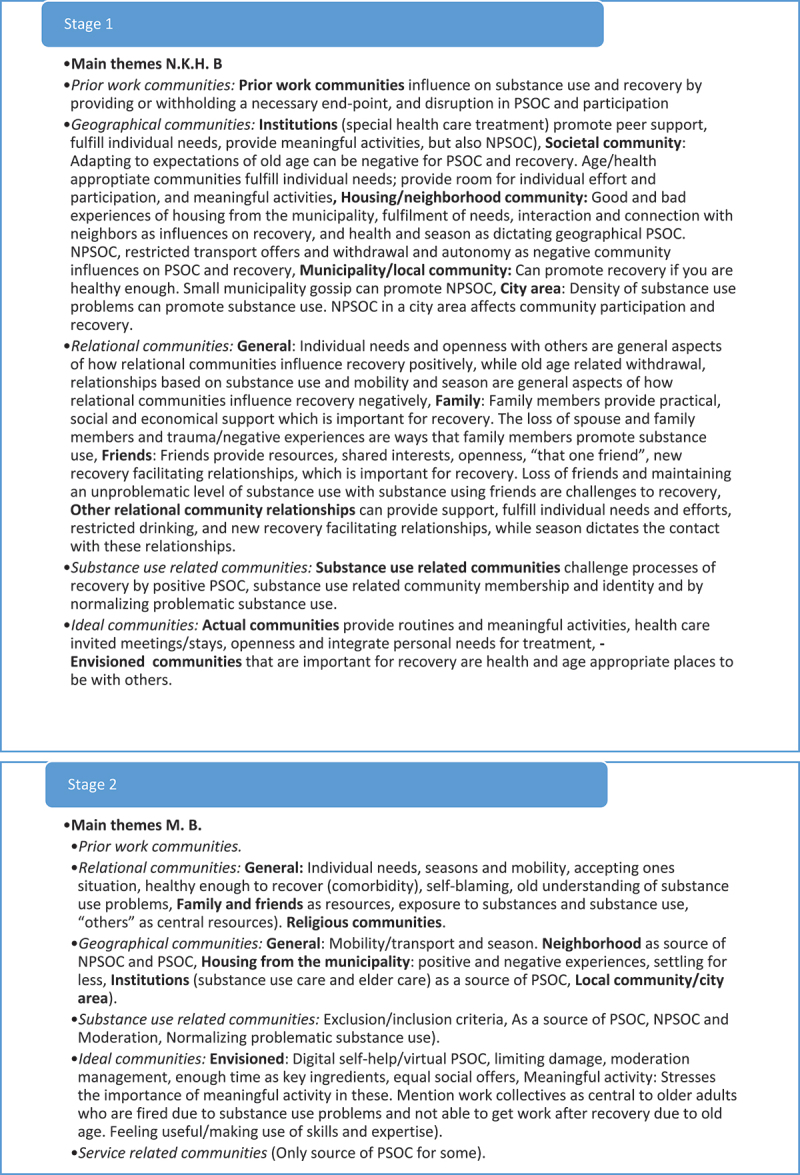
(Continued).

**Figure 1b.**
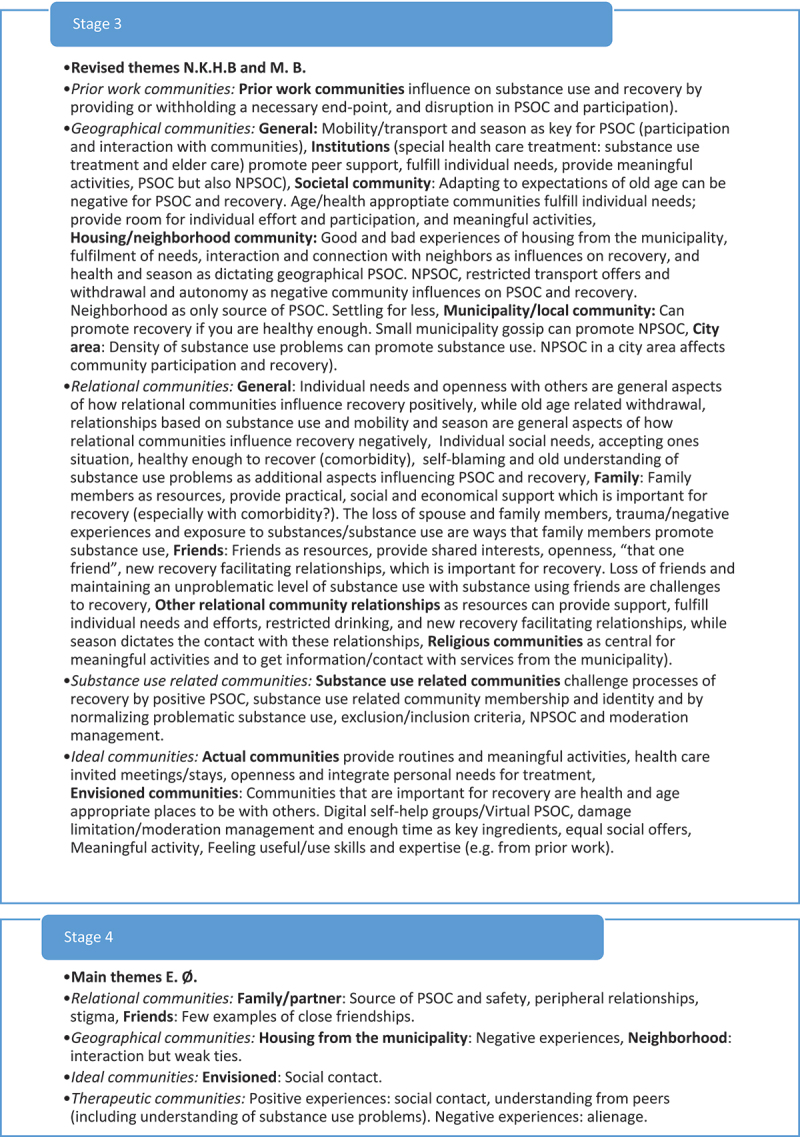
(Continued).

**Figure 1.**
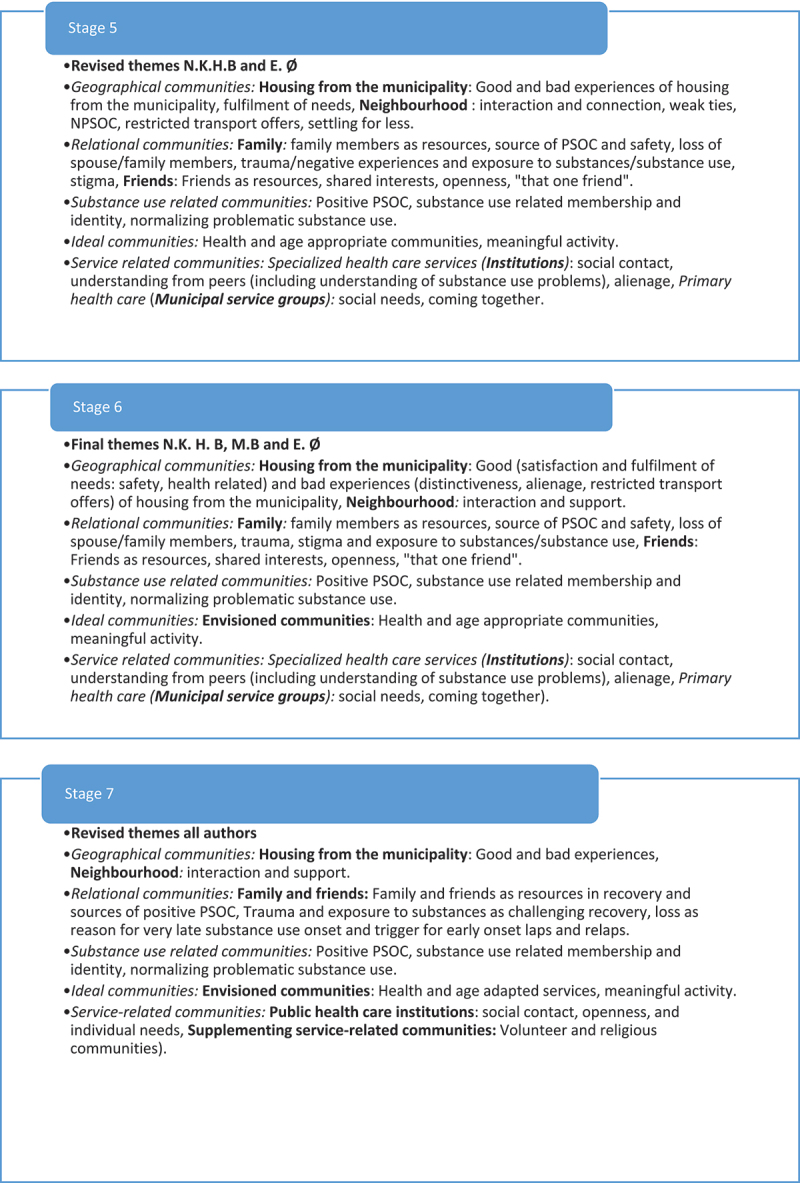
Theme development.

### 7 stages of analysis

As in former MPSOC and recovery studies (Bahl et al., 2019, 2022), we applied deductive, reflexive, and collaborative thematic approach consisting of several stages to analyse the material.

Prior to the current study, the first and second authors undertook a preliminary inductive analysis for a report to the Directorate of Health. In this analysis, it became evident that “family, communities and networks” were central themes in the material. Thus, it was decided to apply the MPSOC concept in a more in-depth and broad analysis of older adults’ descriptions of their communities and their role in recovery.

In the *first and second stages,* the first, second and third authors did their individual reflexive thematic analysis. The first and second authors analysed the entire material of 23 interviews. Deductive line-by-line coding was done in NVivo 12 (QSR International Pty Ltd). Consistent with recommendation for analysis including peer researchers (Pettersen et al., 2019) we then limited the material (of 572 pages) that the peer researcher (third author) coded. 10 interviews were selected based on coding density from the first author’s coding. The selection criteria secured the inclusion of interviews from the three different contexts. The remaining 13 interviews were read and included in his production of themes.

The *third stage* involved a collaborative analysis between the first and the third author and was conducted via Zoom Meetings (Zoom Video Communications, Inc.). Themes from the individual thematic analysis were shared, discussed, and revised. The two authors’ themes overlapped, but the peer researcher’s themes supplemented the first author’s themes with some additional nuances (see Figure 1). Comparing the themes, one may say that the first author’s themes were more theoretically bound: concerned with PSOC and communities in a broad manner, while the peer researcher’s themes emphasized the user perspective and one-to-one relationships in a larger degree. The themes were collaboratively revised to new themes including sub-themes which the two authors agreed upon (see Figure 1).

In the *fourth and fifth stages,* the first and second authors did a second collaborative analysis where they presented their themes (the first author presented the revised themes from the first collaborative analysis to the second author). A new round of discussion, and revision of themes was done until there was an agreement on how to include themes and sub-themes from each of the analysis (see Figure 1). The second author largely specified the themes, by the inclusion of strict community definition, having to go beyond one-to-one experiences of belonging and connection. However, the second authors themes also included sub-themes which nuanced the themes brought to the second collaborative analysis.

In the *sixth stage*, all three authors collaborating on the analysis met via Zoom Meetings) for a final collaborative analysis to finalize the themes for the article. In this analysis, the main discussions concerned which themes to move forward with for the article. Meetings for collaborative analyses were recorded by Zoom, audio, and Teams, allowing the first author to check arguments and decisions made in the meetings. In addition, a document summarizing notes for each step of the analysis as well as a log was written during the analytical process. These are available upon request.

Finally, the final and *seventh stage* of the analysis consisted of a traditional final step of reflexive thematic analysis: writing the report. In this final process, all authors collaborated in revising the themes for the article.

## Findings

Five community references made up the final over-arching themes (see Figure 1): relational, geographical, substance use related, ideal and service related. These communities facilitated and challenged the hard work to maintain PSOC and going through a recovery process in an old age complicated by substance use problems (see Table II).

**Table II.** Overview of community elements participants described as influencing their recovery processes.

Communities	Elements promoting potential for recovery	Elements representing challenges to recovery
1. Relational (family, partners and friends)	Social resources (e.g., information about services, initiating and maintaining contact with services, practical and emotional support in contact with services). Openness about substance use problems. Sources of positive PSOC.	Trauma and exposure to substances. Loss of partner and friends.
2. Geographical (Housing from the municipality and neighbourhood)	Housing from the municipality: Stable housing with appropriate standard and transport offers. Neighbourhood: Everyday positive PSOC, support in situations of illness or help with everyday tasks.	Housing from the municipality: Exposure of substance use, alienage to housing from municipality as a young community and frustration over unsatisfied needs for mobility (restricted transport offers).
3. Substance use-related communities		Positive PSOC Substance use related membership and identity. Normalizing problematic substance use
4. Ideal (envisioned communities)	Support in community participation. Housing adapted to health situation. Peer persons. Meaningful activities.	Lack of information about available services and users rights.
5. Service-related communities (Public health care institutions, religious and volunteer communities)	Elder care institutions: Fulfilment of physical and social needs. Specialized treatment of substance use problems: Openness, respect for individual needs. Religious and volunteer communities: Positive PSOC, new friends, purpose, someone to collaborate with and get support from in the recovery process.	

All quotes have been translated by a professional translator to English, with some small grammatical adjustments for clarity. Participants are represented with codes indicating their gender (F for female, M for male), age, and residential region (E=East, W=West, C=Central) (see Table I for additional information).

### Theme 1: relational communities

Relational communities were the community type most frequently addressed. For several of the participants, the relationships to relational communities such as family and friends were described as distant, fragmented and complex. Also, low frequency of social interaction was a characteristic for those who experienced PSOC with family and friends. A few of the participants described a close relationship with “that one friend” as essential to their PSOC. A small portion of the sample (3) said that they did not have contact with anyone in their everyday life. This part of the sample did not share any information other than that they did not have anyone:

I3: … no, you don’t have kids? How about friends or some other network?

F73 (C22): no, they’re dead, most of them.

I3: really… that must be hard for you.

F73 (C22): yeah. but when you get to be a certain age, that’s how it is, I guess.

We will now describe the sub-themes identified for the themes “family” and “friends”, reflecting family members’ and friends assistance and challenge to recovery.

#### Family and friends as resources in recovery and sources of PSOC

Family and friends are suggested to be important resources in recovery from substance use in old age (Morgan et al., 2011). All of the 23 Norwegian participants addressed the nuclear or extended family members either in a positive or negative manner with respect to their recovery process. The most central sub-theme was *family and friends as resources in recovery and a source of positive PSOC, here illustrated by a 65-year-old participant:*

F65 (C10): … I think many people who fall outside the system don’t get them (the services) … I don’t think I could’ve managed on my own. You have to have them around. Both my kids were there all day. To begin with.

I1: And that’s a lot of work.

F65 (C10): “Yeah.”

Different family relationships were described as central resources in contact with services, ranging from children, partner, siblings, cousins and even more distant relatives like children of their cousins. Both practical and emotional support were fulfiled in relation to services received by these relationships. Examples of practical support were help with documents, managing everyday tasks in periods of sickness (e.g., getting mail, walking the dog and helping with moving) as well as private economy.

With respect to services the practical support included calling services for help and maintaining contact with services:

I4: Yes. You told me a bit about the various services you receive, and how it was for you to get in touch with them when you needed to … Do you mostly use your phone?

M73 (E13): Well, yeah, but I don’t call. My husband calls for me…I don’t really like speaking on the phone..but they’re always very polite. Things always work out when he calls them.

Emotional support was typically described as being there by you side in in contact with services:

I4: Yes. How about your relatives, say your sister, who you said has been involved in your follow-up – has she been important to you?

M76 (E14): Yeah, she has … so, I do have her support. Not that she’d ever let me down, you know.

I4: Mm. So there’s always someone who’s there for you.

M76 (E14): Yes.

Furthermore, openness with family and friends about their substance use problem was identified as a key element enabling these two communities to support recovery:

I2: … could you tell me a bit more about … what’s helped you keep off the drink?

M71 (W4): … yeah, you could say that it’s mainly because I feel more secure when I’ve been [clearing her throat] open, or fairly open, about these things with my kids and family and such … even my grandchildren … when they were old enough, you know, and, yeah, I feel it’s helped to be open about things …

The importance of openness to recovery was also evident in descriptions of long periods of problematic substance use as a result of hiding one’s problems and thus restricting these potentially helping resources from any reaction.

F65 (C10): I didn’t talk about it to anyone. Not my GP, not my closest friends. I have two really close friends. They said they didn’t understand the problem. They said I never smelled of alcohol.

Some of the participants weren’t lucky to have any family that supported them in their recovery. Some of them, however, described “that one particular friend” had been with them for years being an important recovery resource:

M68 (C11): She’s (a friend) been here, and at meetings, both with me and without me.

I1: Has anyone else been involved in your treatment? Your family for instance?

M68 (C11): Well, they know about it, they came to see me at the clinic. But that’s all really.

I1: So it’s mainly your friend who’s been actively involved?

M68 (C11): Yes.

I1: What has that meant to you?

M68 (C11): Well, I guess it’s the reason I’m still here, talking to you today.

In addition to the central and crucial role family and friends played as resources facilitating recovery, these communities were at the same time described also as a *source of PSOC*; as someone to share interests with as well as ups and downs in life.

As can be seen from this sub-theme relational communities such as family and friends can be important resources in later life recovery processes: in initiating recovery, supporting and enabling recovery, and providing positive PSOC for those who are lucky enough to have such social relationships in old age.

#### Trauma and exposure to substances as challenging recovery

Although family and partners were described as central resources for several of the participants’ recovery processes, there was also a dark side to these relationships. Among these experiences were prior traumatic experiences with family members and partner:

I1: Is it anxiety..? Oxazepam, does it help to calm you down?

M69 (C19): I’m having a few problems with that … But I’m fairly calm now. But, of course, the mental problems, like traumas, yeah, I still have them. Had them since I was a kid. Difficult childhood … all because of my crazy mum.

As described, age of onset is central for understanding community belonging and participation. Several participants in the current study described family-related traumatic experiences in the childhood as important for early age of onset of their substance use problems:

M69 (C19): … some people have had to live with it (substance abuse problems) since childhood. Whereas others get drawn into the scene when they’re adults, because of conflicts, bullying at work … or problems with their partners. Things can go wrong. But often it’s because of things that have happened in the past, in childhood, such as … beatings, or violence …

I1: Yes. Or because you come from a family with substance abuse problems?

M69 (C19): Yeah, there’s a lot of that . it has scarred me.

Exposure to substance use was a sub-theme identified among both family and friends. As described, early onset alcoholism might for some be related to genetic predispositions (Neve et al., 1997). This also reflected the participants’ descriptions of family influence.

M70 (C20): The drinking, yeah… that’s a long story…. my Mum drank herself to death … and my Dad tried to do the same …

When it came to friends and early exposure to substance use, the informants described belonging or “getting with the crowd” as related to early onset of substance use:

M77 (E3): There have been times when I’ve drunk too much. When I was young, it was easy to get in with the wrong crowd, where everyone drank … I once had a friend staying at my house. Sometimes, to cope with his drinking, I tried to keep up with him. I’ve drunk a lot over the years, just to fit in with the crowd …

For some of the participants, distancing oneself (a key dimension of NPSOC) from these life-long friend relationships was positive for their recovery.

#### Loss, very late substance use onset and early onset laps or relapse

Loss of partner and friends represent central psychosocial transitions in old age that is related to very late onset drinking and later life relapse (Dar, 2006; Foster et al., 2021). Several of the older adult participants had experienced the death of partner, family and friends. Loss of spouse was typically described as related to late onset of problematic use of alcohol:

I1: When you think back to your drinking problems … did they start after you stopped working? Did that sort of…

M76 (C9): No, I guess it kicked off after my husband died…. After that, enjoying a glass of wine just wasn’t enough, I started to drink more and more.

Importantly, those participants who had very late onset described loss of partner as a *cause* for the development of their problem, while the participants who had an early onset of problematic substance use described the loss of friends in old age as a *trigger* of laps or relapse.

### Theme 2: geographical communities

The participants described geographical communities such as *housing from the municipality* and their *neighbourhood* as influencing their recovery. Generally, few of the participants had contact with members of these communities, but those who did, addressed these communities as affecting their PSOC and everyday life either in a positive or negative manner. Interestingly, season and physical mobility were described by several of the participants as determining participation in geographical communities in general. A nuance in the material illustrating this, was the fact that the participants’ descriptions of only meeting neighbours when the season made it possible:

F73 (E13): … so I don’t really have anything else (in my life) than my neighbours who I run into sometimes. We sit down here and have a chat … that’s something we do.

I4: Both in summer and winter?

F73 (E13): No, mainly in summer. That’s when people are outside and can sit down for a while.

I4: You don’t visit each other at home?

F73 (E13): No, we don’t visit each other. It’s not something we do.

With the restricted opportunity to maintain geographical PSOC in Norway in winter, help with transportation was important for the participants’ recovery.

#### Housing from the municipality: good and bad influences

Housing from the municipality has been identified as mainly experienced as influencing recovery in a negative way in emerging adult and adult age (Bahl et al., 2019, 2022). Over-all, the older adult participants in this study, seemed to have a nuanced view of these geographical communities, including both *positive and negative experiences*. The positive experiences included satisfaction with the level of noise from neighbours and the standard of the housing. Importantly, as addressed by the following participant, this kind of housing could be crucial to one’s self-esteem and well-being:

I5: How’s your accommodation?

M66 (E16): It’s really good. I have a council … a council flat [INAUDIBLE]. It’s really nice. It’s the first time I’ve felt like a proper human being, really… living in a flat and all, wow! When we . tell me how you live, and I’ll tell you who you are, it’s like that isn’t it. Having a place to live, your own flat, means the world to me … I was so happy when she (a council worker) said I’d get a nice council flat. And yes, it was true, that’s what I got. I’m really happy about that!

On the other side, several participants also had negative experiences from this type of housing detrimental to their recovery. Some of these included central dimensions of NPSOC: distinctiveness towards other residents (or substance abusers as a group). Alienage to housing from municipality as a “young community” and frustration over unsatisfied needs for mobility (restricted transport offers) were additional NPSOC-related elements of negative experiences.

Consider this example, illustrating the importance of mobility for PSOC and recovery:

F68 (E5): I, I wasn’t happy there [SIGHING]… It was so lonely …where they moved me to … .I got so depressed that I tried to [GESTURES], which I’ve never done before.

I5: You tried to take your own life?

F68 (E5): Yeah, … well. I waited for hours before they came … in the morning … but I’ve got my TT card now (a card for people with mobility problems who are unable to use ordinary public transport) and now I get some friends visiting…X was a bit far though [for them to come visit] …

Taken together, this sub-theme suggests that the older adults in recovery from substance use problems have a nuanced understanding of housing from the municipality. Moreover, stable housing satisfying needs for silence and an appropriate standard as well as transport offers are described as central components of recovery for users of this service. They also indicate that dimensions included in both PSOC and NPSOC are central concepts to understand later life recovery, also in housing from the municipality.

#### Neighbourhood: interaction and support from neighbours

The seven participants who were in contact with their neighbours described the importance of interaction and support from neighbours. For some of these participants, the interaction with their neighbours was the only contact they had with other people in their everyday life:

I4: Do you have any close friends?

F73 (E13): …no, I don’t, just the neighbours that I run into sometimes. We sit down here and have a chat and that … that’s something we do together.

Neighbours were described as a central source of practical support in situations of illness or need for help with everyday tasks (e.g., looking after pets)

In some situations, neighbourhood support could be lifesaving:

M77 (C8): Yeah, I was a feeling a bit dizzy. So they found me, or, it was my neighbours who noticed, and called an ambulance. They saw the pile of newspapers outside my door.

These findings clearly suggest that neighbours can be a central social resource for older adults everyday PSOC and a resource in recovery processes from substance use problems.

### Theme 3: substance use related communities

Substance use related communities can be a source of a positive PSOC, but are still often not positive for recovery (Bahl et al., 2022). Some of the participants described experiences of positive PSOC, substance use-related membership and identity, and normalizing problematic substance use as ways which substance use-related communities challenged their recovery.

#### Positive PSOC

Although positive PSOC is usually considered central to health and well-being (Bahl et al., 2017; Sarason, 1974; Stewart & Townley, 2020), this isn’t necessarily the case when it comes to persons with substance use problems. Some participants described this type of PSOC as challenging their recovery process:

M71 (W4): … but it was a community, it was sociable … really, really good like that … You want something else, to get out of it (the community) too, but it’s difficult to do something else when you’re so used to being with others with drug or booze problems …

Central to this positive PSOC was the experience of membership and identity in substance use-related communities.

#### Substance use related membership and identity

Recovery from substance use is a social transformation, which entails a change in community membership and social identity (Bahl et al., 2022; Bathish et al., 2017; Groh et al., 2007; Mawson et al., 2015). Several participants described a membership in substance use-related communities despite recovery: F66 (E15): *You see where I feel at home. It’s not difficult to understand, I feel at home among users and drinkers. Even though I haven’t touched either for a long time.*

This type of membership was often experienced as challenging the recovery process, requiring some sort of social management, such as watching out what you drink and not coming off as rude.

From the participants’ descriptions, it seemed as if this kind of membership and identity was founded on which substance you had problems with and age of onset:

M69 (C19): … It’s like that, the drinkers keep away from those who shoot up. And the other way round, those who inject want to stay off the booze.

I1: But why do people keep their distance? Is it because you’re different types of people, or does it have to do with the effect of the substances.?

M69 (C19): Well, we are quite different, because some have had to live with it since childhood, while others fall into the scene when they’re adults, because of conflicts, bullying at work, relationship problems or whatever.

#### Normalizing problematic substance use

Recognizing that one has a substance use problem is essential for the initiation of a recovery process. Substance use-related communities were described as challenging recovery by normalizing what was considered problematic substance use for some of the participants:

M71 (W4): … it’s been quite good being out there (in the drug and alcohol environment) … but most people can’t control themselves when it gets too much (drinking or drug use) and. Also … it has sort of become common to use a lot (of drugs or alcohol) …

### Theme 4: ideal communities

In earlier investigations on community influence on recovery, we have identified ideal communities as a central part of emerging adult and adult participant’s concept of PSOC and recovery (Bahl et al., 2019, 2022). In this study, we identified descriptions of actual ideal communities (including positive, creative and meaningful activities with others) as well as envisioned ideal communities (communities that one imagines that one should have had to recover) have been central parts of these communities (Bahl et al., 2019, 2022). However, actual communities overlapped greatly with service-related communities and those descriptions are represented in the theme “Service related communities”. This overlap makes sense given that the purpose of service-related communities is to promote recovery. As such, consistent with quality practice in thematic analysis to avoid overlapping themes (Braun & Clarke, 2022), we chose to restrict this theme to *envisioned communities*. Two sub-themes were identified for the older adult participants envisioned ideal communities, reflecting their needs for recovery: (a) Health- and age-appropriate communities, and (b) meaningful activities.

#### Health- and age-adapted services

As introduced, older adults with substance use problems often have age- and health-related challenges which are important to consider with respect to later life recovery. When asked about what the participants ideally should have had to recover, several participants mentioned the need for services which made it possible for community participation given their health and age:

I2: … but if you could see things from this side of the fence, say from a GP’s or other service’s point of view … what could they do to make it (recovery) easier?

M71 (W4): Well, it’s like you were saying about having a contact person in the council who deals with the social stuff … I’d like to get more out of life, but my health, both physical and mental, is stopping me … and, yeah, doing things together with others maybe, uhm … if you’re not strong enough to manage things all by yourself …

Housing adapted to health was also mentioned as an envisioned need for the participants’ recovery.

M68 (W6): …also, when it comes to help from the council, I was really hoping to get specially adapted housing from the council, because I’m suffering from [name of illness], in short, it means that I’ve got inflammation in all the nerves in my body … so, at times, I have quite a lot of pain … that’s the most important (to kick the addiction).

In addition to services supporting community participation and health appropriate housing, there were examples in the material illustrating the need for peers in communities: that is, persons who had own experience in potential challenges in recovery processes from substance use problems.

This sub-theme illustrates that supporting community participation, health appropriate housing and peer community members are central elements which older adults see as ideal for later life recovery. Another important element was meaningful activities.

#### Meaningful activities

Meaningful activities have been recognized as an essential component of recovery processes from problematic substance use for emerging adults and adults (Bahl et al., 2022; Emiliussen et al., 2017; Nordaunet & Sælør, 2018; Veseth et al., 2022). Several of the older adults participants described different meaningful activities as desired with respect to their health and recovery in old age. Examples were rather diverse, reflecting different individual interests in painting, philosophizing and discussing, hiking and boat trips. Importantly, having support to get in touch with and do these meaningful activities was key to recovery:

M71 (W4): … of course age plays a part, if you feel you can’t be useful or don’t have a lot to contribute. That’s perhaps the hardest part, wish I could’ve gone hiking in the mountains or by the sea. Even when I was using, I managed to stay clean when I was on a boat and out at sea, fishing or hiking in the mountains or other places, it was sociable. But that’s all gone now that I can’t really get around much.

Taken together, this sub-theme illustrates meaningful activities as highly individual and, once again, that older adults with substance use problems is a heterogenic group. Importantly, the sense of meaning in everyday life seemed to depend on service availability. This dependency made the possibility of a meaningful day rather fragile, where unavailability of one service could result in loneliness.

### Theme 5: service-related communities

In the current data, there were several descriptions of experiences of PSOC and recovery in institutions offering elder care and interdisciplinary specialized substance use treatment. In addition, PSOC and recovery was addressed with respect to supplementary service-related communities: religious and volunteer communities.

#### Public health care institutions

Public health care in Norway offers two types of institutions for older adults with substance use problems: primary (municipal) institutions offering care for the elderly and specialized health care institutions offering interdisciplinary specialized treatment of substance use problems. Generally, however, long periods of residency are needed for PSOC to evolve (Bess et al., 2002), and this was evident in the participants’ descriptions too. PSOC was usually described with reference to institutions offering long-time care for the elderly. These communities were described as important for the participants’ recovery as they fulfilled the needs for support in maintaining physical health as well as for daily social contact. For some of the participants residing in elder care institutions, the social contact with other residents was the only source of PSOC in their everyday life:

I1: Could you tell me a bit about your everyday contact with other people? With neighbours, family, friends?

M77 (C8): Here, I talk to … the lady over there on the terrace, her name is X … Then there’s her in the mobility scooter, she’s on the second floor. They’re the most sociable ones, who I talk to the most. I have some contact with X too …

I1: Does he live here too?

M77 (C8): Yeah, he lives here too.

Stays in *interdisciplinary specialized substance use treatment* were generally shorter (less than 3 months) compared to residency in elderly care institutions. However, those offered polyclinic services maintained a relationship to this service for years. In the communities within this institution (similarly to relational communities), openness was described as important for recovery:

I1: Can you tell me a bit more about the things you think work really well?

F65 (C10): The openness, that you can talk about absolutely anything. Has a lot to do with their professional confidentiality of course. You don’t need to hide anything. You can be yourself, for better or worse.

In addition, respect for individual needs (e.g., autonomy, social needs, and boundaries) was central for recovery in these service-related communities. Of these needs, the need for autonomy was the most frequently mentioned:

I4: Do you find that things get too much sometimes?

F73 (E13): … everyone around you wants to support you and is telling you to stop. and…it got too much for me so at one point I just said no to everything. I put a stop to everything … I just couldn’t take it … the feeling of not having your own life…everyone wanted to tell me how to live my life. I just couldn’t take it…I’m quite independent … I’d had enough.

#### Supplementing service-related communities: religious and volunteer communities

Two supplementary types of service-related communities were identified as important for the participants PSOC and recovery: Religious and volunteer communities. Among the descriptions of these communities were descriptions of belonging to communities founded upon religious values: Salvation Army, anonymous alcoholics and “De norske lenker” *(an NGO for alcohol misuse)*, and experiences of PSOC in a volunteer community (“The soup van”). These communities were central for the participants’ recovery in several ways; by offering community, new friends, a purpose, and someone to collaborate with and get support from in their recovery process. We will shortly provide one illustrating example with respect to De norske lenker:

I4: … what do you think about the help you’ve received.how has it affected your life situation?

M76 (E14): Yeah, I’ve got to go back to Lenkene (De norske lenker). There I got the support I asked for. I can get help there.

I4: Yes. And you’ve got friends for life?

M76 (E14): Yeah. Someone I can go to. A place where I can go.

## Discussion

Our findings confirm and supplement earlier findings and conceptualizations about the role different communities influence later life recovery processes. When it comes to family relationships, our findings *confirm* these relationships as important resources for support in recovery processes (Groh et al., 2007; LaBarre et al., 2021) and the importance of openness for these relational communities to support recovery processes (Emiliussen et al., 2017). Moreover, the findings mirror earlier results about the important interaction between persons’ age of onset, different substance use problems and community membership for later life recovery (Dar, 2006; Emiliussen et al., 2017; Foster et al., 2021). For example, participants with very late onset of alcohol problems described loss of partner as strongly challenging their recovery. There were also stories told by those with early onset of alcohol, confirming an association between early onset and family and friends’ substance use (Groh et al., 2007) as well as loss of friends and heavy drinking in old age (Dar, 2006).

Additionally, some findings *supplement* earlier conclusions. In example, our findings supplement current knowledge about family and friends as sources of general support in recovery from alcohol problems (Dar, 2006; Groh et al., 2007). Our findings suggest that friends and family may provide both practical and emotional support in service assisted recovery from several types of substance use problems in later life. Furthermore, we identified that substance use-related communities promoted a substance use identity, normalization of problematic substance use, and positive PSOC (see Bahl et al., 2019, 2022 for similar findings in adult and emerging adult age), also in old age. Community identity seemed to be a central factor in the interaction of age of onset, type of substance use problem and recovery in later life. Furthermore, the findings illustrate that meaningful activities fulfiling a broad spectre of personal interests are essential for recovery (Bahl et al., 2019, 2022; Emiliussen et al., 2017; Nordaunet & Sælør, 2018; Veseth et al., 2022), also in later life recovery. The findings also generalize earlier findings of transport as a crucial physical capital for community participation (Brekke et al., 2021) in old age. This physical capital may be particularly important for the recovery of older adults with restricted mobility and who reside in more distant areas. Finally, several older adult participants with substance use problems were active and adaptive in their personal recovery processes, striving to maintain PSOC in available communities such as their home, neighbourhoods, and religious and volunteer communities. However, there were also older adults going through recovery processes without the support of anyone. These findings supplement the current view many hold of this “group”; too often primarily subjected to ageism and stigma (Emiliussen et al., 2017; LaBarre et al., 2021; Morgan et al., 2011; Satre et al., 2004).

### So, what is new about the findings?

To our knowledge, this is the first study investigating older adults own accounts of how different communities are experienced as influencing their later life recovery processes. Thus, this study’s findings can be considered as new. However, we would like to highlight what we consider the most central analytical conclusions that can be drawn from the findings.

First, and foremost, this study demonstrates MPSOC as a core dimension in later life recovery. Thus, this study adds an important peace to the puzzle of MPSOC as a dimension in recovery across the life-span (see Bahl et al., 2019, 2022, for studies on MPSOC and recovery in emerging adult and adult years).

Second, the findings provide new insights about the contra-intuitive character of MPSOC in later life recovery. According to the findings, there are instances where positive PSOC in fact can be harmful for recovery and NPSOC on the other hand can facilitate recovery. Thus, although positive PSOC has a positive connotation and NPSOC a negative one, one should not assume them to be so with respect to later life recovery.

Furthermore, as far as we know, this is the first study describing the role volunteer and religious communities have in recovery from substance use in later life. Our findings then suggest that older adults find PSOC and recovery not only in public services, but also in available religious and volunteer communities. Doing so they find peers, new friends, a purpose, and someone to collaborate with and get support from in their personal recovery process.

Finally, the findings provide new contextual insights: The health care system, family orientation and meaning systems emphasizing own responsibility and effort within the sociocultural context were evident in the older Norwegian adults’ accounts about recovery. Thus, community influence on later life recovery has to be treated as a context-sensitive matter.

### Strengths, limitations and future research

Qualitative research uses various criteria for valid knowledge production which include: “sensitivity to the context”, “commitment and rigour”, “coherence and transparency”, and “impact and importance” (Yardley, 2015). We will now highlight this reflexive thematic study’s limitations and strengths with respect to these criteria; also providing suggestions for future research.

*Sensitivity to the context* concern two types of contexts: the context of existing literature about the subject being studied, and the socio-cultural context of the participants (Braun & Clarke, 2013; Yardley, 2015). In this study we have used a broad conceptual framework to gain knowledge about the role different communities’ play in the recovery of older adults. We have also used available literature on the subject to place the findings in a relevant context. Furthermore, we have presented the socio-cultural context of the participants and made analytical conclusions with respect to this context where it has been relevant. Despite these strengths, the small empirical literature about the role communities play in later life recovery from problematic substance use which exists, may have restricted the analytical conclusions of the study. The socio-cultural context and healthcare system in Norway, moreover, are also rather unique in a global perspective, thus restricting the transferability of the findings. Future studies should therefore follow up our findings with respect to other sociocultural contexts.

*Commitment and rigour* are demonstrated by showing that the analysis has been conducted with satisfactory breadth and/or depth to provide added insight to the subject researched (Tracy, 2010; Yardley, 2015). The breadth and depth of this study can be seen regarding the broad theoretical framework, as well as the presented three different perspectives applied in the in-depth deductive and collaborative reflexive analysis (see Figure 1). To our knowledge then, this study is the first utilizing a collaborative design including a peer-researcher in the investigation of any subject related to older adults with substance use problems. Moreover, a heterogenic sample of participants with different substance use problems, ages, age of onset, community relationships, interests, different stages of recovery processes, and from three different residential contexts took part in the study (see Table I). However, the collaborative approach adopted was very time demanding process requiring the researchers to go back-and-forth between multiple perspectives and themes. Including such a broad sample may have resulted in shallower analyses compared to, e.g., analysing the material with respect to sub-groups; for example understanding central group differences, such as gender differences identified in younger age groups or homeless individuals with substance use problems (see Brown et al., 2015; Dar, 2006; Pahwa et al., 2019; Tucker et al., 2020). Thus, despite “starting out” broadly, our approach restrict the transferability of the findings to other sub-groups of older adults with substance use problems. It should also be mentioned that we were not able to include more than 7 women with alcohol and medicine problems. This, most likely, restricted our understanding of nuances in older adult woman’s experiences of community influences on recovery from these two substance use problems. It also means that the experiences of older adult women recovering from illegal substance use problems are not represented in the material. However, the collaborative approach is likely to have enhanced the reflexivity and interpretative depth, compared to a one or two perspective approach. Future studies should develop further knowledge and suggestions for collaborative reflexive thematic approaches so this way of triangulation and user involvement in research can be used more often. Including an older adult peer researcher may enhance the validity in future studies on older adults’ experiences. Moreover, although recruitment through services and use of gift cards were two strategies securing a fairly large number of participants (23) from a heterogenic population difficult to get in touch with, this strategy resulted in a somewhat biased sample of only three participants currently using substances. Future research then should investigate other sampling strategies for recruiting older adults with substance use problems, still using substances.

*Coherence and transparency* deals with the study’s clarity and power: expressing to the reader accurately what was done and why. There should be a good fit between the theoretical approach, research question, methods used and the interpretations of the data (Braun & Clarke, 2022; Yardley, 2015). So far, we consider to have sufficiently described the fit between the MPSOC theoretical framework, the research question, the choice of semi-structured interviews, and the collaborative deductive reflexive approach to analyse and interpret the data. We have also provided the reader with detailed information about the participants in this study (see Table I) and the analytical process behind the production of knowledge (see Figure 1). In addition, we have pointed out that documents from the analysis as well as a log are available upon request, making our approach even more transparent.

Finally, this study’s *impact and importance* are particularly demonstrated by the need and request for knowledge about how to promote recovery in later life complicated by substance use (Gfroerer et al., 2003; Johannessen et al., 2016), specifically on how to provide broader and better care of older adults by the inclusion of significant others (e.g., family careers) in their recovery (Bahl, Landheim, et al., 2021; Johannessen et al., 2015; Morgan et al., 2011). Thus, implications of our findings—that is the way that the findings make a difference—will now be elaborated.

### Practical Implications for substance use services

There is a pressing need for substance use services tailored to older adults and consequently also knowledge about how to promote recovery in later life (Gfroerer et al., 2003; Johannessen et al., 2016; Morgan et al., 2011). Based on impact and importance of the findings, we will now make some suggestions for how substance use services can promote PSOC and recovery in later life.

First, our findings suggest that older adults’ recovery processes are highly personal and heterogenic (e.g., with respect to community relationships, interests, individual needs, type of problematic substance use, age and age of onset). Thus, we strongly suggest that personalized treatment and clinical pathways for older adults are tailored according to age of onset, type of substance use problem, personal interests, and social resources available.

Second, the findings illustrate that PSOC and recovery among older adults with substance use problems are multi-dimensional matters including multiple communities (relational, geographical, substance use related, ideal and service related) as well as affective states (PSOC and NPSOC). Furthermore, we advise that MPSOC dimensions for older adults are mapped and assessed at the initiation of substance use recovery (see Bahl, Landheim, et al., 2021 for suggestions) so that preventive and promotive approaches to recovery can be developed. Mapping and assessing MPSOC dimensions are crucial to gain central information to promote recovery facilitating elements and prevent barriers to later life recovery (see Table II for examples). Having mapped and assessed individual MPSOC, service professionals thereby are likely to be better equipped in collaborating with older adults in promoting recovery facilitating community elements, thereby preventing community elements destructive to their recovery (e.g., by asset-based community development approaches and dialogical network approaches).

Third, religious and volunteer communities offered some of the participants a community, new friends, a purpose, and someone to collaborate with and get support from in their recovery processes. Thus, we would also like to underline here the importance of public health services for older adults to collaborate with these communities. Such support is particularly important for those older adults who do not have the assistance and benefit of recovery promoting elements in their communities.

Fourth, the findings indicate that restricted transport offers less physical mobility. Lack of help and assistance to”get out” clearly represent obstacles for older adults’ PSOC and their recovery. Thus, it is important that older adults have available transport offers, as well as health adapted (e.g., 1^st^ floor apartments) and age appropriate (e.g., silent, proper standard and residents from same cohort) housing.

Finally, to promote recovery in old age it is necessary to get in touch with the older adults with high discrepancy between service need and utilization—particularly older adults with mild-to-moderate drinking problems, older adult women with AUD or pharmaceutical opioid addiction and those with rather few social resources (Gfroerer et al., 2003; Johannessen et al., 2016; Morgan et al., 2011; Rhodes et al., 2018; Tucker et al., 2020). Thus, offering assertive community treatment tailored for different groups of older adults is central for reaching these groups of older adults.

To sum up, building community relationships is a key to promote recovery processes from substance use also for older adults. Thus, adapting and strengthening the culture of belonging and service approaches to community are important ongoing circles for improving older adults’ various needed healthcare services.

## Concluding remarks

Being involved, to feel sense of belonging and have a meaningful life without substance use are key aspects of recovery from substance use problems (Bahl et al., 2019, 2022; Granfield & Cloud, 2001; Groh et al., 2007; Johannessen et al., 2015; Laudet, 2007; Mayberry et al., 2009; Moore et al., 2018; Mudry et al., 2019; Panel, 2007). Our findings suggest that later life processes of recovery have to be understood as multidimensional (influenced by multiple community references and affective states: PSOC and NPSOC) and heterogenic (influenced by interactions between age of onset, type of substances and available recovery facilitating communities, fulfilment of needs and meaningful activities). Furthermore, the findings suggest later life recovery require individual management and support through later life psychosocial transitions (e.g., loss of spouse or friends and reduction in physical mobility). Both recovery facilitating community relationships (e.g., supportive family, friends and neighbours) and services fulfiling personal needs (e.g., available transportation, meaningful activities) are described as important ingredients in a meaningful later life without substances.

To conclude, the findings *extend* and *nuance* current understandings of later life recovery as personal and social processes with the added complexity of age and substance use such as: decline in social networks and community participation due to the death of spouse or partner, loss of friends, retirement from the work community, and deterioration in physical mobility (LaBarre et al., 2021; Morgan et al., 2011), high likelihood of depression, shame, loneliness and isolation (Emiliussen et al., 2017; LaBarre et al., 2021; Morgan et al., 2011; Satre et al., 2004; Yarnell et al., 2020). To sum up, this study extends current understanding by demonstrating that later life recovery may be more multidimensional and heterogenic than previously assumed: Additional communities, affective states and personal factors seem to be important nuances to understand later life recovery. Thus, the findings illustrate that MPSOC can be a useful concept, with central practical and theoretical implications for the applied and theoretical field of later life recovery.
